# Potent prion-like behaviors of pathogenic α-synuclein and evaluation of inactivation methods

**DOI:** 10.1186/s40478-018-0532-2

**Published:** 2018-04-18

**Authors:** Airi Tarutani, Tetsuaki Arai, Shigeo Murayama, Shin-ichi Hisanaga, Masato Hasegawa

**Affiliations:** 1 Department of Dementia and Higher Brain Function, Tokyo Metropolitan Institute of Medical Science, 2-1-6 Kamikitazawa, Setagaya-ku, Tokyo, 156-8506 Japan; 2 Department of Biological Science, Tokyo Metropolitan University, Minami-osawa, Hachioji, Tokyo, 192-0397 Japan; 3 Department of Neuropsychiatry, Division of Clinical Medicine, Faculty of Medicine, University of Tsukuba, 1-1-1, Tennodai, Tsukuba, Ibaraki, 305-8576 Japan; 4 Department of Neuropathology, Tokyo Metropolitan Geriatric Hospital and Institute of Gerontology, 35-2 Sakaecho, Itabashi-ku, Tokyo, 173-0015 Japan

**Keywords:** α-Synuclein, Prion-like propagation, Seeds, α-Synucleinopathy, Strains, Inactivation

## Abstract

The concept that abnormal protein aggregates show prion-like propagation between cells has been considered to explain the onset and progression of many neurodegenerative diseases. Indeed, both synthetic amyloid-like fibrils and pathogenic proteins extracted from patients’ brains induce self-templated amplification and cell-to-cell transmission in vitro and in vivo. However, it is unclear whether exposure to exogenous prion-like proteins can potentially cause these diseases in humans. Here, we investigated in detail the prion-like seeding activities of several kinds of pathogenic α-synuclein (α-syn), including synthetic fibrils and detergent-insoluble fractions extracted from brains of patients with α-synucleinopathies. Exposure to synthetic α-syn fibrils at concentrations above 100 pg/mL caused seeded aggregation of α-syn in SH-SY5Y cells, and seeded aggregation was also observed in C57BL/6 J mice after intracerebral inoculation of at least 0.1 μg/animal. α-Syn aggregates extracted from brains of multiple system atrophy (MSA) patients showed higher seeding activity than those extracted from patients with dementia with Lewy bodies (DLB), and their potency was similar to that of synthetic α-syn fibrils. We also examined the effects of various methods that have been reported to inactivate abnormal prion proteins (PrP^Sc^), including autoclaving at various temperatures, exposure to sodium dodecyl sulfate (SDS), and combined treatments. The combination of autoclaving and 1% SDS substantially reduced the seeding activities of synthetic α-syn fibrils and α-syn aggregates extracted from MSA brains. However, single treatment with 1% SDS or generally used sterilization conditions proved insufficient to prevent accumulation of pathological α-syn. In conclusion, α-syn aggregates derived from MSA patients showed a potent prion-like seeding activity, which could be efficiently reduced by combined use of SDS and autoclaving.

**Electronic supplementary material:**

The online version of this article (10.1186/s40478-018-0532-2) contains supplementary material, which is available to authorized users.

## Introduction

Intracellular accumulations of abnormal protein aggregates are common but defining neuropathological features of many neurodegenerative diseases. The distributions and spreading of these pathological proteins are closely correlated with clinical symptoms and progression [9, 49]. Recently, it has been suggested that the prion-like behavior of abnormal proteins may account for the onset and progression of neurodegenerative diseases [21, 62]. A growing body of evidence supports the idea that template-mediated amplification and intracerebral transmission of abnormal proteins are the main mechanisms by which pathological proteins spread along the neural circuits in the brain, although the molecular mechanisms of cell-to-cell transmission remain to be fully clarified.

α-Synucleinopathies, which include Parkinson’s disease (PD), dementia with Lewy bodies (DLB) and multiple system atrophy (MSA), are characterized by accumulation of misfolded α-synuclein (α-syn) aggregates in neuronal and/or glial cells, and various pathological phenotypes and clinical symptoms are observed for each disease [22]. In PD and DLB, α-syn pathologies are mainly observed in neurons in the form of Lewy bodies (LBs) and Lewy neurites (LNs) [4, 56], whereas glial cytoplasmic inclusions (GCIs) are seen in oligodendrocytes in MSA [61]. The abnormal α-syn observed in brains of patients is accumulated as fibrous or filamentous forms with cross-β structures [54, 55], existing in phosphorylated and partially ubiquitinated states [18, 24]. These abnormal α-syn species exhibit seeding activity to induce prion-like conversion, detergent-insolubility and protease-resistance of endogenous α-syn [38], being similar in these respects to the infectious forms of prion protein (PrP^Sc^) causing Creutzfeldt-Jakob disease (CJD) and bovine spongiform encephalopathy [45].

α-Syn is a natively unfolded protein of 140 amino acid residues, which is localized in synaptic termini at relatively high concentration [33]. Although its physiological function has not been fully clarified, it appears to be involved in the regulation of SNARE complex and in dopamine production [1, 6, 12, 13]. Disease-linked missense mutations and multiplication of the *SNCA* gene encoding α-syn have been reported in familial forms of α-synucleinopathies, indicating that structural changes and overexpression of α-syn protein are involved in the development of synucleinopathies [42].

Recombinant soluble α-syn proteins purified from bacterial cells expressing α-syn form amyloid-like fibrils that are morphologically and physicochemically similar to those observed in patients’ brains in vitro, upon shaking at 37 °C for a few days [14, 51]. These synthetic α-syn fibrils can act as seeds and induce seeded aggregation of α-syn in cultured cells or primary cultured neurons, as well as in rodent brains [25]. Intracerebral inoculation of synthetic α-syn fibrils induces phosphorylated and ubiqutinated α-syn pathologies not only in transgenic (Tg) mice overexpressing human α-syn, but also in wild-type (WT) mice [31, 32, 36]. In particular, Tg mice overexpressing mutant human α-syn develop lethal central nervous system (CNS) disorder after being inoculated with fibrils [37]. It has also been shown that brain homogenates or insoluble fractions extracted from brains of patients with α-synucleinopathies induce α-syn pathologies in animal brains [5, 36, 47, 63]. In addition, recent studies have suggested that α-syn strains with distinct conformations exist, which is a characteristic of prions. Synthetic α-syn fibrils formed under different physiological conditions in vitro showed distinct seeding activities and cytotoxicities in cultured cells and rat brains [8, 43]. Furthermore, MSA brain extracts exhibit distinct infectivity compared to PD or control brain extracts in human embryonic kidney (HEK) 293 cells expressing mutant A53T α-syn fused to yellow fluorescent protein and in TgM83 hemizygous mice expressing mutant A53T α-syn [46, 66].

These experimental demonstrations of prion-like propagation show that abnormal α-syn can trigger self-templated amplification of abnormal forms and spread throughout the brain. On the other hand, there is no report or evidence as yet to indicate that infection of pathogenic α-syn between individuals can occur, or that onset of α-synucleinopathy can occur following exposure to contaminated environments. It is also unclear to what extent materials contaminated with pathogenic α-syn pose a risk of secondary infection to patients, clinicians and researchers. These are clearly important issues, because iatrogenic transmission of PrP^Sc^ from human tissues derived from patients with CJD (e.g. dura mater transplants and growth hormone treatments) or from surgical instruments contaminated with PrP^Sc^ is well documented [11]. Therefore, we need to examine whether iatrogenically acquired abnormal α-syn may cause synucleinopathy or accelerate clinical symptoms. Here, we examined this question by characterizing the seeding activities of synthetic α-syn fibrils and abnormal α-syn extracted from brains of patients with MSA and DLB.

We have already established experimental models for seeded aggregation of α-syn using SH-SY5Y cells, as well as in vivo models in WT rodents and primates inoculated intracerebrally with synthetic α-syn fibrils [36, 41, 53]. Our previous study showed that the size of the amyloid-like fibrils is the key factor that determines the efficiency of seeded aggregation and propagation in these models [58]. In this study, we further investigated the seeding activities of these pathogenic α-syn species, focusing on how much pathogenic α-syn is required to seed the formation of intracellular aggregates of transiently expressed or endogenous normal α-syn beyond the capacity of maintenance mechanisms, such as lysosomal, proteasomal and autophagy-mediated protein degradation systems, to remove them. We also investigated whether the abnormal α-syn aggregates derived from MSA and DLB patients’ brains show distinct prion-like properties characteristic of each disease, compared to synthetic α-syn fibrils, in our models.

It is also important to consider inactivation methodology. It has been reported that a commercially available alkaline cleanser, a hydrogen peroxide solution containing Cu^2+^ ions, and 1% sodium dodecyl sulfate (SDS) are effective for removing pathogenic α-syn adhering to surgical materials and laboratory instruments [7, 60]. In addition, BioHOCl effectively reduced the seeding activity of pathogenic α-syn derived from brains with Lewy-body pathology, as determined with a real-time quaking induced conversion (RT-QuIC) assay [26]. On the other hand, formalin fixation of tissues containing α-syn pathology derived from patients with synucleinopathies and from symptomatic Tg mice had little effect on seeding activity or infectivity [50, 65]. Notably, we found that α-syn aggregates derived from MSA patients’ brains (MSA-syn) exhibited much higher seeding activity than those from DLB cases. Fortunately, however, MSA-syn and other abnormal α-syn could be efficiently reduced by combined use of SDS and autoclaving. The present findings, together with previous work, suggests that safety measures to destroy pathogenic α-syn should be mandatory in hospitals and laboratories.

## Materials and methods

### Expression and purification of α-syn protein

*Escherichia coli* BL21 (DE3) was transfected with bacterial expression plasmid pRK172, a construct containing human WT or mouse α-syn that lacks cysteine due to mutagenesis of codon 136 (TAC to TAT) [34], and the expressed protein was purified as described [39]. Protein concentrations of α-syn were determined by reverse-phase HPLC as described [35].

### Preparation of recombinant α-syn fibrils

Purified recombinant α-syn proteins (5 mg/ml) containing 30 mM Tris-HCl (pH 7.5), 10 mM DTT and 0.1% sodium azide were incubated for 7 days at 37 °C in a horizontal shaker (Taitec) at 200 rpm, then ultracentrifuged at 113,000×g for 20 min at 25 °C. The pellets were washed with saline and ultracentrifuged as before. The resulting pellets were collected as α-syn fibrils and resuspended in 30 mM Tris-HCl (pH 7.5). The fibrils at a concentration of 2 mg/ml were fragmented using a cup horn sonicator (Sonifier® SFX, Branson) at 35% power for 180 s (total 240 s, 30 s on, 10 s off), then aliquoted and cryopreserved. For electron microscopy, α-syn fibrils (0.2 μg) were dropped on carbon-coated 300-mesh copper grids (Nissin EM) and incubated for 3 min. After removal of surplus material, the fibrils were negatively stained for 3 min with a drop of 2% sodium phosphotungstate and dried. Electron micrograph images were obtained with a JEOL JEM-1400 electron microscope (JEOL).

### Ethics statement

Postmortem brain tissues, which had been neuropathologically confirmed as MSA or DLB, were obtained from the Brain Bank for Aging Research in Tokyo Metropolitan Geriatric Hospital & Institute of Gerontology, (Tokyo, Japan). The study protocol was approved by the ethics committees of Tokyo Metropolitan Geriatric Hospital and Tokyo Metropolitan Institute of Medical Science. All methods were performed in accordance with the relevant guidelines and regulations. All brain tissues used in this study were anonymized.

All mice were housed at the Tokyo Metropolitan Institute of Medical Science (TMiMS) in a facility in compliance with Guidelines for Proper Conduct of Animal Experiments (Science Council of Japan). Experimentation followed TMiMS Animal Care and Use Committee approved protocol #17018 in compliance with Guidelines for Proper Conduct of Animal Experiments (Science Council of Japan).

### Preparation of sarkosyl-insoluble fractions from patients’ brains

For each case, a brain sample (0.5 g) was homogenized in 20 volumes (*w*/*v*) of A68 buffer (10 mM Tris-HCl pH 7.5 containing 10% sucrose, 0.8 M NaCl, 1 mM EGTA) and incubated for 30 min at 37 °C, after addition of sarkosyl (final concentration: 2%). Brain homogenates were centrifuged at 9460×g for 10 min at 25 °C, then ultracentrifuged at 113,000×g for 20 min at 25 °C. The pellets were washed with saline and ultracentrifuged as before. The resulting pellets were collected as sarkosyl-insoluble fractions of patients’ brains, resuspended in 30 mM Tris-HCl (pH 7.5) by sonication for 15 s, and used for introduction into cultured cells or for inactivation treatments. Resuspended fractions were centrifuged at 1000 g for 5 min. The supernatants were used for stereotaxic inoculation.

For immunoblotting analysis, sarkosyl-insoluble fractions were added to SDS-sample buffer and boiled for 3 min. The samples were separated by 12% SDS-PAGE. Immunoblotting with mouse monoclonal antibody PSer129 (1:1000) directed against α-syn phosphorylated at Ser129 [18] and other anti-α-syn antibodies, including syn131-140 (1:2000, Cosmo bio), LB509 (1:1000) and Syn102 (1:1000) was performed as described [52]. LB509 and Syn102 were kind gifts from Dr. Takeshi Iwatsubo. Phosphorylated tau was detected with monoclonal anti-Tau antibody T46 (1:1000, Thermo Fisher Scientific).

### Quantification of α-syn by immunoblotting

The concentrations of α-syn in sarkosyl-insoluble fractions extracted from human brain samples were determined by immunoblotting. The standard curve was generated using serial dilutions of recombinant phosphorylated human α-syn monomer. Phosphorylated α-syn was detected with mouse monoclonal anti-PSer129 antibody. The band intensities of immunoblots were quantified using ImageQuant TL.

The concentrations of α-syn expressed in WT mice were calculated by immunoblotting as below. The standard curves were generated using recombinant mouse α-syn. C57BL/6 mouse brains were homogenized in A68 buffer and centrifuged at 10,000 g for 5 min. The supernatant were collected as A68-soluble fraction and immunoblotted with polyclonal anti-mouse α-syn antibody (1:1000, Cell Signaling Technology).

### Immunoelectron microscopy

Sarkosyl-insoluble fractions extracted from MSA and DLB patients’ brains were dropped onto carbon-coated nickel grids (Nissin EM). The grids were immunostained with an anti-phosphorylated α-syn rabbit monoclonal antibody EP1536Y (Abcam, 1:200) and a secondary antibody conjugated to 5 nm gold particles (BBI Solutions, 1:50) as described [23]. Electron micrograph images were recorded with a JEOL JEM-1400 electron microscope (JEOL).

### Cell culture, transfection of plasmids and introduction of pathogenic proteins into cells

Human neuroblastoma SH-SY5Y cells were maintained at 37 °C in 5% CO_2_ in Dulbecco’s modified Eagle’s medium (DMEM)/F12 medium (Sigma-Aldrich) supplemented with 10% fetal calf serum, penicillin-streptomycin glutamine (Gibco), and MEM nonessential amino acids solution (Gibco). Cells were cultured to 40–50% confluence in collagen-coated 6-well plates and transfected using X-tremeGENE 9 (Roche Life Science) with pcDNA3 encoding human WT α-syn (1.5 μg) according to the manufacturer’s instructions. After transfection of plasmids, cells were incubated for 6–8 h, and pathogenic proteins (2 μl) were introduced using MultiFectam (Promega) according to the manufacturer’s instructions. Transfected cells were incubated for 3 days.

### Preparation of sarkosyl-insoluble fractions from transfected cells and immunoblotting

Transfected SH-SY5Y cells were collected and extracted with 1 ml of 1% sarkosyl in A68 buffer. Cell extracts were sonicated for 15 s on ice. After incubation for 30 min at 37 °C, cell extracts were ultracentrifuged at 113,000×g for 20 min at 25 °C. The supernatants were removed and collected as sarkosyl-soluble fractions, then the pellets were washed with 30 mM Tris-HCl (pH 7.5) and ultracentrifuged as before. The resulting pellets were collected as sarkosyl-insoluble fractions, resuspended in 30 mM Tris-HCl (pH 7.5) and sonicated for 15 s. Sarkosyl-insoluble and -soluble fractions were added to SDS-sample buffer and boiled for 3 min. The protein concentrations of samples were determined with a Pierce BCA Protein Assay Kit (Thermo Fisher Scientific). Immunoblotting was performed with mouse monoclonal anti-PSer129 antibody (1:1000) and polyclonal antibody syn131–140 (1:2000, Cosmo Bio). In immunoblotting, monoclonal anti-α-tubulin (1:1000, Sigma) was used to obtain a loading control. All experiments were performed at least three times. The band intensities of immunoblots were quantified using ImageQuant TL. The ID_50_ was calculated by the Spearman-Karber method based on the band intensities.

### Immunofluorescence microscopy

Transfection of plasmids and introduction of pathogenic proteins were conducted as described above, using SH-SY5Y cells grown on coverslips. After incubation for 3 days, cells were fixed with 4% paraformaldehyde and treated with an anti-phosphorylated α-syn rabbit monoclonal antibody EP1536Y (1:1000, Abcam) and the secondary antibody (anti-rabbit IgG-conjugated Alexa-568, Invitrogen) as described [57]. The cells were mounted and analyzed using a BZ-X710 fluorescence microscope (Keyence).

### Mice

C57BL/6 J mice were purchased from CLEA Japan, Inc.

### α-Syn inoculation into mouse brains and immunohistochemistry

α-Syn samples (5 μl) were inoculated into striatum (A-P: 0.2 mm; M-L: − 2.0 mm; D-V: − 2.6 mm) as described [35]. The inoculum was injected at a rate of 5 μl per min and the needle was kept in place at the injection site for 3 min or more. At 3 months after inoculation, mice were anesthetized with isoflurane and killed by decapitation. Brains were fixed with 10% formalin neutral buffer solution (Wako) and sectioned at 50 μm with a Leica VT1200S (Leica). Immunohistochemistry with an anti-phosphorylated α-syn rabbit monoclonal antibody EP1536Y (1:1000, Abcam) was performed as described [53]. α-Syn pathologies were observed and recorded with a BZ-X710 fluorescence microscope (Keyence). Immunoreactivity for pS129 α-syn was quantified using images of coronal sections from four different regions (frontal cortex, striatum, amygdala, substantia nigra); 4–7 sections per animal for each region were used for quantification. PS129-positive cells were counted in 20× (striatum, amygdala and substantia nigra) or 10× (frontal cortex) images using BZ-H3C Hybrid Cell Count Software (Keyence).

### Inactivation and protease treatments

Synthetic α-syn fibrils (2 mg/ml) and sarkosyl-insoluble fractions extracted from MSA patients’ brains were incubated in saline or in 0.1% or 1% sodium dodecyl sulfate for 1 h at room temperature. Pathogenic α-syn in saline or 0.1% or 1% SDS was also incubated at 100 °C for 3 min, 120 °C for 20 min or 134 °C for 20 min. Incubations at 120 °C and 134 °C were performed using an laboratory autoclave, LSX-300 (Tomy). For protease K treatment, inactivated synthetic α-syn fibrils were treated with 5 μg/ml protease K at 37 °C for 30 min. The reaction was stopped by boiling, and samples were analyzed by immunoblotting with polyclonal antibody syn102–116 (1:2000) directed against a synthetic peptide (residues 102–116) (Cosmo Bio).

### Statistical analysis

The DLB and MSA data presented in Fig. 3b were analyzed using Welch’s modified t-test. All other data were analyzed using a one-way ANOVA followed by Dunnett’s post hoc test. In both cases, a *P* value < 0.05 was regarded as statistically significant.

## Results

### Seeded aggregation induced by synthetic α-syn fibrils in SH-SY5Y cells

**Fig. 1:**
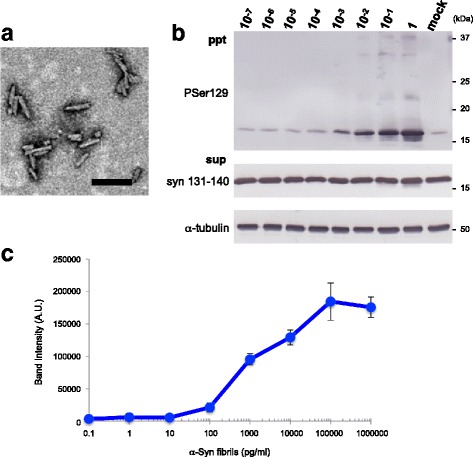
Seed-dependent α-syn aggregation induced by serial dilutions of synthetic α-syn fibrils in SH-SY5Y cells. **a** Electron microscopy of human α-syn fibrils after sonication for 180 s. Negatively stained short fibrils less than 100 nm in size were observed. Scale bar, 100 nm. **b** Serial 10-fold dilutions of human α-syn fibrils (2 μl) were introduced into SH-SY5Y cells transiently expressing human WT α-syn in the presence of the transfection reagent. Immunoblot analysis of sarkosyl-insoluble fractions (ppt) and sarkosyl-soluble fractions (sup) extracted from mock-transfected cells or cells transfected with human α-syn fibrils in the range of 2 μg (1) to 0.2 pg (10^− 7^) are shown. Phosphorylated α-syn was detected with anti-phosphorylated α-syn PSer129 antibody. α-Syn was detected with anti-syn 131–140 antibody **c** Quantification of phosphorylated α-syn accumulated in SH-SY5Y cells exposed to serial dilutions of synthetic human α-syn fibrils. Band intensities from the immunoblot analyses shown in **b** were measured. The results are expressed as means ± SEM (*n* = 3).

First, we investigated the minimum amounts of pathogenic α-syn required to induce seeded aggregation and accumulation of phosphorylated α-syn in SH-SY5Y cells expressing untagged human wild-type (WT) α-syn. Serial dilutions from 10^− 1^ to 10^− 7^ of 1 mg/ml (70 μM) synthetic human WT α-syn fibrils (Fig. 1a) were sonicated for 3 min and then introduced into SY5Y cells as previously reported [41]. After 3 days, the sarkosyl-insoluble fraction was prepared from the transfected cells and accumulation of phospho-α-syn was analyzed by immunoblotting with PS129 antibody (Fig. 1b). The results of quantitation of the insoluble phosphorylated α-syn are shown in Fig. 1c. Accumulation of phospho-α-syn was detected in cells treated with α-syn fibrils diluted to 10^− 3^, but no increase was observed in cells treated with dilutions from 10^− 4^ to 10^− 7^. The relative amounts of insoluble α-syn formed in cells were increased concentration-dependently by addition of α-syn fibrils at dilutions from 10^− 1^ to 10^− 4^, but the amount was saturated at more than 10^− 1^ dilution (Fig. 1c). Based on protein determination by immunoblotting, the ID_50_ (50% infectious dose or seeding activity) per 2 μL was calculated to be 10^2.91^ (± 0.51) by the Spearman-Karber method [64]. These results show that exposure of SH-SY5Y cells transiently expressing human α-syn to synthetic human α-syn fibrils at a concentration of above 100 pg/mL can induce seeded aggregation of the intracellular α-syn.

### Characterization of insoluble α-syn extracted from brains of patients with α-synucleinopathies

**Fig. 2:**
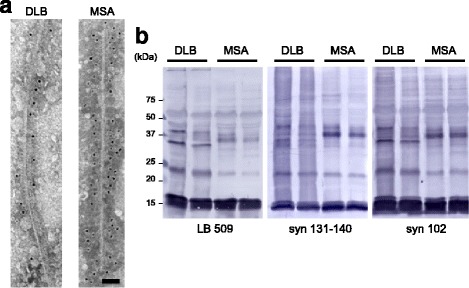
Characterization of α-syn aggregates extracted from brains of synucleinopathy patients. **a** Immunoelectron microscopy of sarkosyl-insoluble fractions extracted from DLB (left) and MSA (right) patients’ brains. Electron micrographs show fibrous structures positive for PSer129 antibody EP1536Y, that were labeled with secondary antibody conjugated to 5 nm gold particles. Scale bar, 50 nm. **b** Immunoblot analyses of sarkosyl-insoluble fractions prepared from brains of synucleinopathy patients. Sarkosyl-insoluble α-syn (22, 29 and 37 kDa in DLB, and 22 and 32 kDa in MSA) were detected by LB509 (left), anti-syn 131–140 (center) and Syn 102 (right) antibodies

Next, we investigated the prion-like properties of α-syn aggregates derived from postmortem brain tissues of patients with α-synucleinopathies. To examine whether there are structural and biochemical differences between α-syn aggregates in DLB and MSA, we performed immuno-electron microscopy and immunoblotting of sarkosyl-insoluble fractions extracted from patients’ brains. PS129-positive α-syn fibrous structures with 5–10 nm diameter and 0.1 to 1 μm length were observed in both cases, as previously reported [55] (Fig. 2a). Fibrils derived from MSA and labeled with PS129 antibody appeared slightly different from those derived from DLB. A majority of the fibrils were twisted with 80–100 nm periodicity in MSA, while thinner, straight fibrils were observed in DLB (Fig. 2a). Fibrous structures from DLB were subtle curvy and longer compared to those from MSA (Fig. 2a). Immunoblot analysis of sarkosyl-insoluble fractions extracted from DLB and MSA patients’ brains with LB509, syn131–140 and Syn102 antibodies showed different banding patterns. All the antibodies detected 22, 29 and 37 kDa α-syn species in DLB, while 22 and 32 kDa α-syn species were detected in MSA (Fig. 2b). It is reported that these bands detected in DLB are ubiquitinated forms of α-syn [2, 24], suggesting that accumulated α-syn shows distinct post-translational patterns in DLB and MSA.

### Prion-like properties in SH-SY5Y cells of α-syn aggregates extracted from patients’ brains

**Fig. 3:**
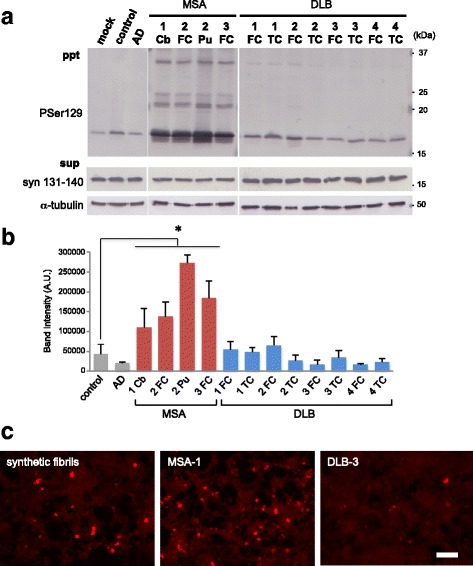
Prion-like properties in SH-SY5Y cells of α-syn aggregates extracted from brains of synucleinopathy patients. **a** Sarkosyl-insoluble fractions extracted from patients’ brains (2 μl) were introduced into SH-SY5Y cells transiently expressing human WT α-syn. Immunoblot analysis of sarkosyl-insoluble fractions (ppt) and sarkosyl-soluble fractions (sup) extracted from mock-transfected cells, and sarkosyl-insoluble fractions from cerebellum, frontal cortex and putamen of 3 MSA cases, frontal cortex and temporal cortex of 4 DLB cases, control brain and an AD case. Phosphorylated α-syn was detected with anti-phosphorylated α-syn PSer129 antibody. α-Syn was detected with anti-syn 131–140 antibody. The α-syn concentrations of Sarkosyl-insoluble fractions derived from human brains are shown in Additional file 1: Table S1A. **b** Quantification of immunoblot analyses shown in A. The results are expressed as means ± SEM (*n* = 3). *P < 0.05. **c** SH-SY5Y cells into which synthetic human α-syn fibrils (2 μg) or sarkosyl-insoluble fractions from cerebellum of a MSA case and temporal cortex of a DLB case (2 μl) had been introduced were fixed and immunostained with PSer129 antibody EP1536Y. Scale bar, 100 μm. Cb: cerebellum, FC: frontal cortex, Pu: putamen, TC: temporal cortex

**Fig. 4:**
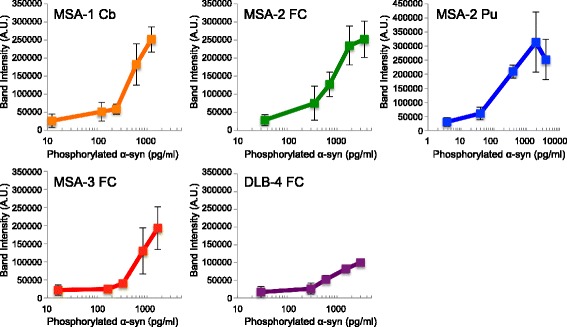
Seed-dependent α-syn aggregation induced in SH-SY5Y cells by serial dilutions of insoluble fractions extracted from brains of patients with synucleinopathies. Serial dilutions of sarkosyl-insoluble fractions prepared from cerebellum, frontal cortex and putamen of 3 MSA cases and frontal cortex of a DLB case (2 μl) were introduced into SH-SY5Y cells transiently expressing human WT α-syn. Quantification of phosphorylated α-syn accumulated in SH-SY5Y cells induced by serial dilutions of pathogenic α-syn derived from brain samples. Band intensities of immunoblot analysis shown in Additional file 4: Figure S3 were measured. The results are expressed as means ± SEM (*n* = 3). Cb: cerebellum, FC: frontal cortex, Pu: putamen, TC: temporal cortex

To examine the seeded aggregation of α-syn in cultured cells, we prepared sarkosyl-insoluble fractions from 3 cases of MSA (cerebellum, frontal cortex and putamen), 4 cases of DLB (frontal cortex and temporal cortex), one case of Alzheimer’s disease (AD) and one control, and analyzed them by immunoblotting with PS129 and anti-tau antibody T46. PS129-positive phospho-α-syn bands were detected in all the sarkosyl-insoluble fractions from both MSA and DLB (Additional file 1: Figure S1A). Pathological-tau (PHF-tau) bands at 60, 64, and 68 kDa together with C-terminal fragments were detected in AD and one case of DLB (Additional file 1: Figure S1A). Pathological tau and α-syn bands were not detected in the control case. The concentrations of phosphorylated α-syn in these sarkosyl-insoluble fractions were calculated from a calibration curve prepared with recombinant phosphorylated α-syn (Additional file 1: Figure S1B and Additional file 2: Table S1A). MSA-2 samples (frontal cortex and putamen) contained high concentrations of α-syn (3.58 and 7.72 ng/μl, respectively), but the other MSA and DLB samples contained lower amounts of α-syn in the range of 1.11~ 1.94 ng/μl (Additional file 2: Table S1A). The same amount (2 μL) of the sarkosyl-insoluble fractions was introduced into SH-SY5Y cells transiently expressing untagged human WT α-syn with Multifectam (Promega), and the cells were cultured for three days. Seeded aggregation in the cells was detected by immunoblotting of sarkosyl-insoluble phosphorylated α-syn. Little or no increase of phosphorylated α-syn was detected in cells treated with α-syn aggregates derived from DLB brains (DLB-syn from frontal and temporal cortexes) compared to those in cells treated with α-syn from control and AD brains (Fig. 3a and b). On the other hand, α-syn aggregates derived from MSA brains (MSA-syn) induced a marked increase of insoluble phosphorylated α-syn (Fig. 3a and b). Phosphorylated α-syn was not derived from the brain samples added to the cells, since no phosphorylated α-syn was detected in the insoluble fraction of SH-SY5Y cells without transient expression of α-syn after transduction with MSA-syn (data not shown). In addition, the accumulation of insoluble α-syn was correlated with the formation of PS129-positive α-syn aggregates in SH-SY5Y cells (Fig. 3c). These results are consistent with previous reports that MSA prions induced fluorescence-labeled α-syn aggregate formation in HEK 293 cells and earlier lethal CNS disorders in TgM83 hemizygous mice, whereas PD prions did not show similar infectivity [46, 66]. Next, we quantitated the prion-like seeding activities of MSA-syn and DLB-syn by using serially diluted samples (1/2, 1/5, 10^− 1^, 10^− 2^ and 10^− 3^) of sarkosyl-insoluble fractions extracted from 3 cases of MSA (cerebellum, frontal cortex and putamen) and 1 case of DLB (frontal cortex). All MSA-syn exhibited high seeding activity at the concentration of 100 pg/ml, whereas DLB-syn exhibited much lower seeding activity at the same concentration (Fig. 4 and Additional file 3: Figure S2). Thus, these results indicated that MSA-syn possesses a strain-specific prion-like character distinct from that of DLB-syn. In addition, no significant differences in the seeding activity were detected among the different brain regions used in this study. MSA-syn has high seeding activity, similar to that of sonicated synthetic α-syn fibrils (Figs. 1c and 4).

### Lewy-like α-syn pathology in WT mice inoculated with serial dilutions of synthetic α-syn fibrils

**Fig. 5:**
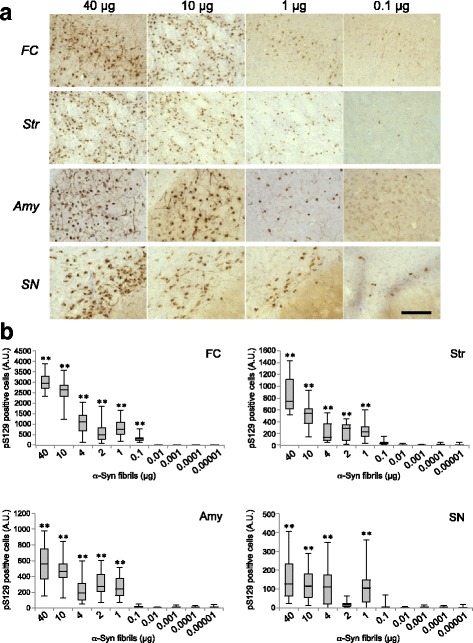
Dose-dependent propagation following introduction of different amounts of synthetic α-syn fibrils in non-Tg mice. **a** Various amounts of mouse α-syn fibrils inoculated into WT mice dose-dependently induced α-syn pathology in frontal cortex, striatum, amygdala and substantia nigra. α-Syn pathology in mouse brains inoculated with 40, 10, 1, and 0.1 μg of α-syn fibrils into striatum at 3 months after inoculation is shown. The numbers of injected mice are shown in Additional file 5: Table S2A. Sections were evaluated by immunohistochemistry with PSer129 antibody EP1536Y. Scale bar, 100 μm. **b** Quantification of α-syn pathology in brains of mice inoculated with different amounts (40 μg to 10 pg) of mouse α-syn fibrils into striatum. The box plots show the number of pS129-positive cells in different regions. One-way ANOVA with Dunnett’s post hoc test was used for multiple comparisons to 10 pg, *P < 0.05; **P < 0.01. FC: frontal cortex, Str: striatum, Amy: amygdala, SN: substantia nigra

We characterized pathogenic α-syn in WT mice by means of the same approach as for SH-SY5Y cells. Series of diluted synthetic mouse α-syn fibrils were prepared and dilutions of 40, 10, 4, 2, 1, 10^− 1^ to 10^− 5^ μg were inoculated into striatum in the right hemisphere of C57BL6 mice. At 3 months after inoculation, phosphorylated α-syn pathology was examined by immunohistochemistry with phospho-α-syn antibody PS129. In mouse brains inoculated with more than 0.1 μg of synthetic mouse α-syn fibrils, Lewy-like pathologies were detected in striatum, frontal cortex, amygdala, substantia nigra and entorhinal cortex, as previously reported [35] (Fig. 5a), whereas inoculation of less than 0.01 μg α-syn fibrils did not induce α-syn pathology. To investigate the correlation between inoculum dose and spreading of α-syn pathology, we quantitated phosphorylated α-syn-positive cells and neurites in striatum, frontal cortex, amygdala and substantia nigra, where relatively abundant, conspicuous, broad-spectrum pathologies were observed in our previous study [58]. The numbers (areas) of phosphorylated α-syn-positive nerve cells and neurites were increased in parallel with the inoculated dose of α-syn fibrils (Fig. 5b). The increases were particularly marked in striatum and frontal cortex near the inoculation site. These results indicate that intracerebral inoculation of at least 0.1 μg of synthetic mouse α-syn fibrils per animal is required to induce Lewy-like pathology in WT mouse brain, in which the concentration of endogenous α-syn was determined to be 37.03 (± 6.71) μg/mL (Additional file 4: Figure S3).

### MSA-syn induced PS129-positive α-syn pathology in WT mice

**Fig. 6:**
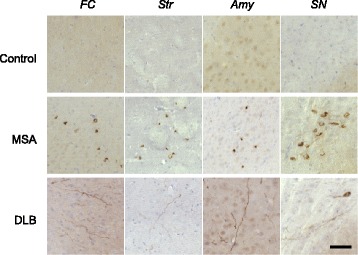
α-Syn strain-specific pathology in non-Tg mice. Inoculation of sarkosyl-insoluble fractions extracted from MSA brain (MSA-2, putamen, 5 μl) into WT mice induced PS129-positive inclusions in frontal cortex, striatum, amygdala and substantia nigra at 3 months after inoculation (middle). Sarkosyl-insoluble fractions extracted from DLB brain (5 μl) induced Lewy-neurite-like α-syn pathology at 9 months after inoculation (lower). Control brain did not induce α-syn pathology (upper). Sections were evaluated by immunohistochemistry with PSer129 antibody EP1536Y. The α-syn concentrations of sarkosyl-insoluble fractions derived from human brains are shown in Additional file 2: Table S1B. The numbers of injected mice are shown in Additional file 5: Table S2C. FC: frontal cortex, Str: striatum, Amy: amygdala, SN: substantia nigra

We next investigated whether these distinct pathogenic α-syn aggregates derived from MSA and DLB induce different types of pathology in WT mice. MSA-syn and DLB-syn were inoculated into the right striatum and α-syn pathology was evaluated by immunohistochemistry with PS129. Inoculation of MSA-syn derived from MSA-2 (putamen), which contained the highest concentration of phosphorylated α-syn, induced PS129-positive inclusions in striatum, frontal cortex, amygdala and substantia nigra at 3 months after inoculation (Fig. 6). α-Syn pathologies in MSA-syn-injected mouse brains did not resemble the GCIs-like pathology observed in MSA, but resembled the neuronal inclusions observed in PD and DLB. DLB-syn induced little α-syn pathology at 3 months after inoculation, and the pathology was mostly Lewy-neurite-like, even at 9 months after inoculation, as previously reported [36] (Fig. 6). No α-syn pathology was observed in mice inoculated with the control brain sample at 3 or 9 months after inoculation (Fig. 6). These results showed that α-syn aggregates derived from brains of patients with MSA and DLB induce distinct pathological forms of α-syn in WT mouse brains, supporting the view that MSA-syn and DLB-syn are distinct α-syn strains.

Thus, we determined the minimum amounts of synthetic α-syn and MSA-syn required for induction of seeded aggregation in SH-SY5Y cells and those of synthetic α-syn fibrils required for prion-like propagation of pathological α-syn in WT mice. In addition, we confirmed that MSA-syn and DLB-syn show distinct morphological and biochemical features and exhibit strain-specific prion-like properties in these models.

### Degradation of synthetic α-syn fibrils by pre-vacuum steam sterilization and SDS

**Fig. 7:**
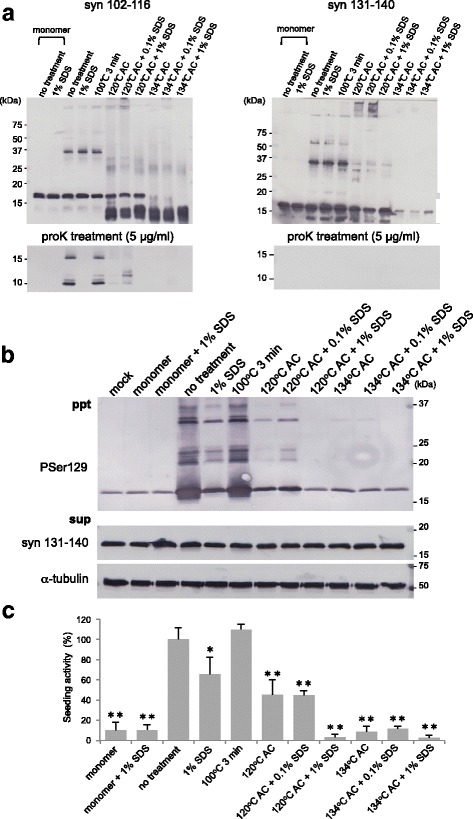
Inactivation of synthetic α-syn fibrils by SDS treatment and autoclaving. **a** Synthetic α-syn fibrils were subjected to various inactivation treatments and analyzed by immunoblotting with Syn 102–116 and anti-syn 131–140 antibodies (upper). Treated α-syn samples were treated with protease K (5 μg/mL) and analyzed by immunoblotting (lower). **b** α-Syn fibrils were subjected to various inactivation treatments (2 μl) and introduced into SH-SY5Y cells transiently expressing human WT α-syn. Immunoblot analysis of sarkosyl-insoluble fractions (ppt) and sarkosyl-soluble fractions (sup) extracted from mock-transfected cells and cells transfected with α-syn monomer and fibrils, and treated with 1% SDS for 1 h at room temperature, or boiled, or autoclaved (AC) at 120 °C with or without 0.1%, 1% SDS, or autoclaved at 134 °C with or without 0.1% or 1% SDS are shown. Phosphorylated α-syn was detected with anti-phosphorylated α-syn PSer129 antibody. α-Syn was detected with anti-syn 131–140 antibody. **c** Quantification of immunoblot analysis shown in **b**. The results are expressed as means ± SEM (*n* = 3). “No treatment” was taken as 100%. One-way ANOVA with Dunnett’s post hoc test were used for multiple comparisons to no treatment, *P < 0.05; **P < 0.01

Having seen that small amounts of synthetic α-syn fibrils and MSA-syn can cause seeded aggregation of α-syn and induce α-syn pathology in vitro and in vivo, we next tested whether several procedures used to inactivate PrP^Sc^ are also able to reduce the seeding activity of pathogenic α-syn. Synthetic α-syn fibrils and MSA-syn were subjected to general sterilization and inactivation procedures for PrP^Sc^, and the residual seeding activities were examined using SH-SY5Y cells and WT mice. First, we tried SDS and autoclave treatments of synthetic α-syn fibrils. Synthetic human WT α-syn fibrils (2 mg/ml; 140 μM) were incubated for 1 h in the presence or absence of 0.1% or 1% SDS at room temperature or boiled at 100 °C for 3 min. Autoclave treatments of α-syn fibrils at 120 °C or 134 °C for 20 min were also conducted in the presence or absence of 0.1% and 1% SDS. The resulting samples were analyzed by immunoblotting with anti-α-syn antibodies. Some degradation bands of ~ 10 kDa were detected with the Syn102–116 antibody after autoclaving synthetic α-syn fibrils (Fig. 7a), suggesting that α-syn was hydrolysed by high-pressure and high-temperature treatments. Moreover, α-syn bands were hardly detectable with the anti-α-syn 131–140 antibody after autoclave treatment at 134 °C, indicating that the α-syn C-terminal region was mostly degraded after the higher-temperature autoclave treatment used in the inactivation of PrP^Sc^ (Fig. 7a). The proteinase K (ProK) resistance of these treated α-syn samples was also examined to characterize the inactivation. ProK-resistant 10 and 15 kDa bands were detected with Syn102–116 in synthetic α-syn fibrils with or without boiling at 100 °C for 3 min, whereas no ProK-resistant band was detected in α-syn fibrils after autoclaving or 1% SDS treatment, although weak bands were observed after autoclaving at 120 °C in the presence and absence of 0.1% SDS (Fig. 7a).

### Seeding properties of synthetic α-syn fibrils in SH-SY5Y cells after inactivation treatments

To test the effectiveness of the inactivation treatments, synthetic human α-syn fibrils after these treatments were introduced into SH-SY5Y cells and the intracellular accumulation of phosphorylated α-syn was examined by immunoblotting with PS129. Seeding activity of α-syn fibrils boiled at 100 °C for 3 min was almost equal to that of untreated fibrils (Fig. 7b and c). The single 1% SDS treatment and the autoclaving at 120 °C in the presence or absence of 0.1% SDS reduced the accumulation of α-syn but 50~ 80% of the seeding activity remained (Fig. 7b and c). On the other hand, the seeding activity was decreased by about 90% compared to that of untreated fibrils after autoclaving at 120 °C in the presence of 1% SDS, or 134 °C in the presence or absence of SDS (Fig. 7b, c and Table 1). In particular, combined treatments with 1% SDS and autoclaving resulted in almost complete abrogation of seeding activity (Fig. 7b, c and Table 1). Notably, the seeding activities of synthetic α-syn fibrils after various treatments corresponded well to the amounts of ProK-resistant bands detected with Syn102–116 and to the degree of degradation of α-syn fibrils (Fig. 7a).

**Table 1:** Inactivation effects of pathogenic α-syn derived from recombinant α-syn protein and MSA patients’ brains in SH-SY5Y cells

Treatment	Synthetic α-syn fibrils	MSA-2 Pu	MSA-3 FC
No treatment	100 (± 11.22)	100 (± 3.38)	100 (± 11.49)
1% SDS	65.5 (± 16.48)	48.6 (± 18.66)	13.8 (± 2.96)
100 °C 3 min	109.7 (± 5.00)	114.2 (± 20.45)	29.5 (± 2.07)
120 °C 20 min	45.3 (± 14.75)	75.1 (± 13.45)	17.5 (± 2.37)
120 °C 20 min/0.1% SDS	44.9 (± 4.45)	not tested	not tested
120 °C 20 min/1% SDS	3.3 (± 2.94)	10.5 (± 2.91)	1.7 (± 1.23)
134 °C 20 min	8.7 (± 5.31)	19.0 (± 2.13)	6.3 (± 0.93)
134 °C 20 min/0.1% SDS	11.5 (± 2.31)	not tested	not tested
134 °C 20 min/1% SDS	2.8 (± 2.76)	3.5 (± 0.95)	6.8 (± 0.25)

**Fig. 8:**
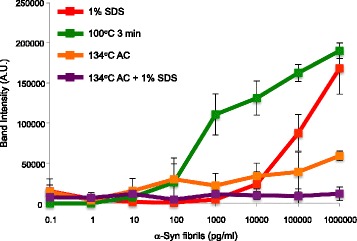
Effects of inactivation treatments of synthetic α-syn fibrils on seeding activity in SH-SY5Y cells. Serial 10-fold dilutions of human α-syn fibrils treated with 1% SDS (red), boiling (green), autoclaving (AC) at 134 °C (orange) or autoclaving at 134 °C with 1% SDS (purple) were introduced into SH-SY5Y cells transiently expressing human WT α-syn. Phosphorylated α-syn accumulated in SH-SY5Y cells exposed to serial dilutions of synthetic human α-syn fibrils were quantified after each treatment. Band intensities from the immunoblot analyses shown in Additional file 6: Figure S4 were measured. The results are expressed as means ± SEM (*n* = 3)

To further investigate their inactivation, synthetic α-syn fibrils treated with 1% SDS at room temperature for 1 h, boiling at 100 °C for 3 min and autoclaving at 134 °C in the absence or presence of 1% SDS, then diluted serially from 10^− 1^ to 10^− 6^, and introduced into SH-SY5Y cells. Synthetic α-syn fibrils after boiling at 100 °C for 3 min retained seeding activity equivalent to that of untreated fibrils, shown in Fig. 1c (Fig. 8 and Additional file 6: Figure S4). The single 1% SDS treatment reduced the seeding activity to about 1/100 (Fig. 8 and Additional file 6: Figure S4). The ID_50_ values per 2 μL of synthetic α-syn fibrils treated with 1% SDS and with boiling were calculated to be 10^2.94^ (± 0.67) and 10^2.13^ (± 0.48), respectively. On the other hand, α-syn fibrils after autoclaving at 134 °C in the absence or presence of 1% SDS showed little seeding activity (Fig. 8 and Additional file 6: Figure S4).

### Inactivation of pathogenic α-syn derived from brains of MSA patients

**Fig. 9:**
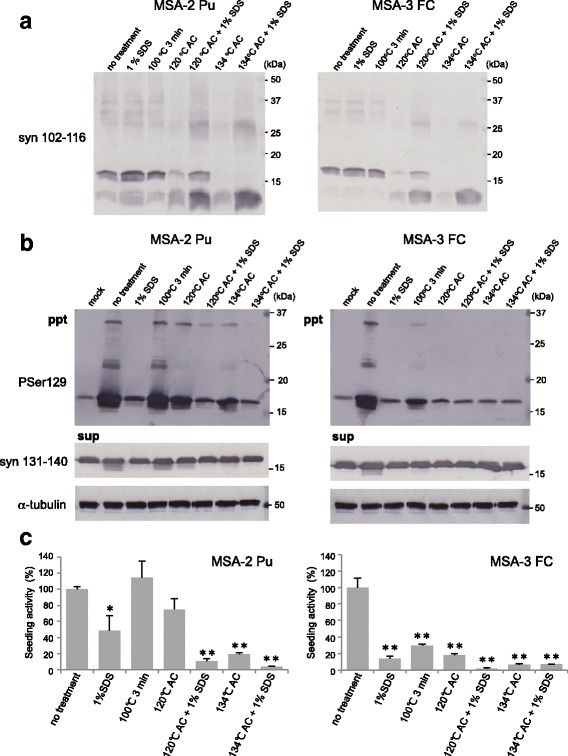
Inactivation of pathogenic α-syn derived from brains of MSA patients. **a** Sarkosyl-insoluble fractions prepared from 2 MSA cases (MSA-2 Pu and MSA-3 FC) after various inactivation treatments were analyzed by immunoblotting with anti-syn 102–116 antibody. **b** Sarkosyl-insoluble fractions extracted from MSA brains after inactivation treatments (2 μl) were introduced into SH-SY5Y cells transiently expressing human WT α-syn. Immunoblot analyses of sarkosyl-insoluble fractions (ppt) and sarkosyl-soluble fractions (sup) extracted from mock-transfected cells or cells transfected with pathogenic α-syn derived from MSA brains and treated with 1% SDS for 1 h at room temperature, boiling, autoclaving (AC) at 120 °C with or without 1% SDS or AC at 134 °C with or without 1% SDS are shown. Phosphorylated α-syn was detected with anti-phosphorylated α-syn PSer129 antibody. α-Syn was detected with anti-syn 131–140 antibody. **c**, Quantification of immunoblot analyses shown in **b**. The results are expressed as means ± SEM (*n* = 3). “No treatment” was taken as 100%. One-way ANOVA with Dunnett’s post hoc test were used for multiple comparisons to no treatment, *P < 0.05; **P < 0.01

We also investigated whether these inactivation treatments are effective for pathogenic α-syn derived from patients with MSA. Sarkosyl-insoluble fractions extracted from 2 cases of MSA (MSA-2, putamen and MSA-3) were treated with 1% SDS, boiling at 100 °C for 3 min, or autoclaving at 120 °C or 134 °C for 20 min in the presence or absence of 1% SDS. Immunoblotting with the Syn102–116 antibody showed marked degradation of insoluble α-syn after the autoclave treatments in the presence and absence of 1% SDS, in agreement with the results for synthetic α-syn fibrils shown in Fig. 7a (Fig. 9a). No apparent difference in the α-syn bands was detected between untreated MSA-syn and MSA-syn after single treatment with 1% SDS or boiling (Fig. 9a). When the treated MSA-syn was introduced into SH-SY5Y cells together with the untreated samples, we found that the seeding activity of MSA-syn from MSA-2 was dramatically reduced by about 90% after autoclaving at 121 °C or 134 °C in the presence of 1% SDS, compared to untreated MSA-syn (Fig. 9b, c and Table 1). Single autoclave treatment at 134 °C decreased the activity by about 80% (Fig. 9b, c and Table 1). The seeding activity was not completely lost after single 1% SDS treatment or autoclaving at 120 °C (Fig. 9b, c and Table 1). Boiling at 100 °C for 3 min had no effect (Fig. 9b, c and Table 1). Interestingly, MSA-syn derived from MSA-3 exhibited greater sensitivity to the inactivation treatments. The seeding activity was partially decreased after boiling at 100 °C for 3 min, and almost abolished after the other treatments (Fig. 9b, c and Table 1). These results suggest that some varieties of pathogenic α-syn from MSA are resistant to inactivation, although differences in the concentration of pathological α-syn in the fractions may also affect the seeding activity. These results showed that the seeding activity of synthetic α-syn fibrils could be efficiently reduced by autoclaving treatment in presence of 1% SDS, and this procedure was also effective to inactivate pathogenic α-syn derived from MSA patients.

### Seeding properties of inactivated pathogenic α-syn in WT mice

**Fig. 10:**
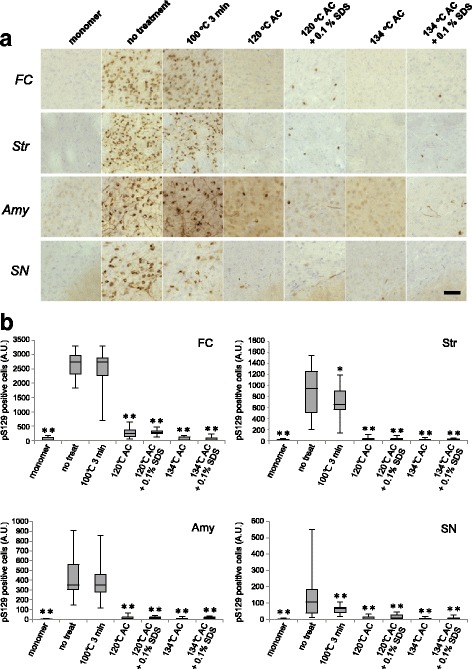
Effects of inactivation treatments of synthetic α-syn fibrils on seeding activity in non-Tg mice. **a** 2 mg/ml mouse α-syn fibrils were exposed to various treatments (1% SDS for 1 h at room temperature, boiling, autoclaving (AC) at 120 °C with or without 0.1% SDS or autoclaving at 134 °C with or without 0.1% SDS), and 5 μl aliquots of the resulting samples were inoculated into WT mice. α-Syn pathologies in frontal cortex, striatum, amygdala and substantia nigra at 3 months after inoculation are shown. The numbers of injected mice are shown in Additional file 5: Table S2B. Sections were evaluated by immunohistochemistry with PSer129 antibody EP1536Y. Scale bar, 100 μm. **b** Quantification of α-syn pathology in mouse brains inoculated into striatum with α-syn fibrils exposed to various treatments. The box plots show the numbers of pS129-positive cells in different regions. One-way ANOVA with Dunnett’s post hoc test were used for multiple comparisons to no treatment, *P < 0.05; **P < 0.01. FC: frontal cortex, Str: striatum, Amy: amygdala, SN: substantia nigra

Finally, we investigated the effects of the inactivation procedures on seeding activity in vivo. Synthetic mouse α-syn fibrils after the inactivation treatments were inoculated into the right striatum of WT mouse brains. At 3 months after inoculation, α-syn pathology was evaluated by immunohistochemistry with PS129. Synthetic α-syn fibrils treated by boiling at 100 °C for 3 min induced Lewy-like pathology almost equivalent to that seen after inoculation of untreated fibrils, as had been found in the cellular model (Fig. 10a and b). Autoclaving of synthetic α-syn fibrils in the presence or absence of 0.1% SDS reduced the seeding activity, and the resulting α-syn pathologies were similar to those of mice inoculated with 0.1 μg of untreated synthetic fibrils, shown in Fig. 2a (Fig. 10a and b). The single autoclave treatment at 134 °C was the most effective treatment to reduce the seeding activity in mice (Fig. 10a and b). We also performed inoculation of synthetic fibrils after autoclaving in the presence or absence of 1% SDS, but the effects of treatments could not be compared because the mouse brains were partially lysed by SDS.

**Fig. 11:**
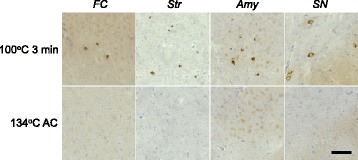
Effects of inactivation treatments of pathogenic α-syn extracted from MSA patients’ brains on seeding activity in non-Tg mice, α-Syn pathology in mouse brains inoculated into striatum with sarkosyl-insoluble fractions extracted from MSA brain (MSA-2, putamen) after boiling or autoclaving (AC) at 134 °C, at 3 months after injection. The numbers of injected mice are shown in Additional file 5: Table S2C. Sections were evaluated by immunohistochemistry with PSer129 antibody EP1536Y. Scale bar, 100 μm. FC: frontal cortex, Str: striatum, Amy: amygdala, SN: substantia nigra

MSA-syn derived from MSA-2 after boiling at 100 °C for 3 min and autoclaving at 134 °C was also inoculated into mouse brains. At 3 months after inoculation, PS129-positive inclusions were observed in mice injected with boiled MSA-syn, as shown in Fig. 5, whereas no pathology was detected in the mice injected with MSA-syn after autoclaving at 134 °C (Fig. 11). Thus, the autoclave treatment at 134 °C reduced the seeding activity of synthetic α-syn fibrils and MSA-syn sufficiently to block induction of α-syn pathology in WT mice.

## Discussion

Many experimental studies on prion-like propagation of pathological proteins, such as α-syn, tau and TDP-43 strongly support the idea that cell-to-cell transmission of abnormal protein aggregates is a central mechanism in the pathogenesis and progression of many neurodegenerative diseases. Recent reports have also suggested that structurally different conformations of α-syn or tau exhibit strain-specific biochemical properties, including differences in the efficiency of propagation and in cellular toxicities. On the other hand, these findings raise the question of whether these pathogenic proteins can act as prions, i.e., infectious agents that may cause the onset and progression of neurodegenerative diseases, when humans are exposed to them. So far, there is no report of transmission of these pathogenic proteins between humans or from animals to humans, except in the case of PrP^Sc^. However, considering the potential consequences of secondary infection to patients, clinicians and researchers, it is essential to establish whether there is actually a risk of iatrogenic or other infection. Therefore, in this study, we examined the ability of pathogenic α-syn proteins to induce prion-like seeded aggregation and to propagate in cellular and animal models. We also investigated the efficacy of various inactivation methods for reducing the seeding activity of these pathogenic α-syn.

First, the seeding activities of serially diluted synthetic α-syn fibrils and α-syn aggregates derived from brains of synucleinopathy patients were characterized in SH-SY5Y cells transiently expressing human WT α-syn, in terms of formation of aggregated and phosphorylated α-syn. In this model, synthetic α-syn fibrils caused seeded aggregation of human α-syn at 7.14 pM and higher concentrations (Fig. 1c). The concentration of human soluble α-syn in the cells was estimated to be 2 μM, suggesting that the synthetic α-syn fibrils could function as seeds at 10^8^ times lower concentration than that of normal α-syn. Further, MSA-syn showed similar seeding activity to the synthetic fibrils, although DLB-syn showed a lower seeding activity (Figs. 3b and 4). The concentration of pathogenic α-syn required for infection was similar to that reported by Prusiner’s group, who detected seeded aggregation by fluorescence measurement in an HEK293 cell model [66]. In the present study, the synthetic fibrils were used after powerful sonication, which enhances their seeding activity, as shown in our previous report. However, MSA-syn showed high seeding activity even without this sonication. This may indicate that there are some structural differences between MSA-syn and the synthetic fibrils. We also found that MSA-syn is morphologically and biochemically distinct from DLB-syn (Fig. 2). It is plausible that these differences reflect the formation of the fibrils in different intracellular environments (oligodendrocytes in MSA and neuronal cells in PD and DLB). Further investigations will be required to understand the roles of different of α-syn strains in the clinical and pathological diversity of α-synucleinopathies.

We then used a WT mouse model to examine the minimum infectious amount, and found that as little as 0.1 μg of synthetic α-syn fibrils could induce Lewy-like pathology of endogenous mouse α-syn (Fig. 5). This is quite large compared to PrP^Sc^, and thus the infectivity of α-syn fibrils as an environmentally acquired prion is expected to be considerably lower than that of PrP^Sc^. The reason for this may be that normal prion protein is localized on the cell surface by a glycosylphosphatidyl-inositol (GPI) anchor, but the intracellular localization of α-syn requires that pathogens should first pass through the cell membrane to reach their target. Lewy-like pathologies in synthetic fibril-injected mice increased in proportion to the inoculation volume, especially around the injection site (Fig. 5). Similar dose-dependent induction of pathology was reported in the inoculation of synthetic tau fibrils into PS19 Tg mice overexpressing mutant human tau (P301S), in which MC1-positive tau pathology increased near the inoculation site when the inoculation volumes were increased [27].

For evaluation of prion-like properties, various methods are available, including RT-QuIC assay, prion-like propagation experimental models using cultured cells, primary cultures and animals, although they show different sensitivities. We analyzed the prion-like seeding activity by detecting formation of intracellular insoluble α-syn in SH-SY5Y cells transiently expressing α-syn or in WT mice expressing endogenous α-syn. In the cells, exposure to synthetic α-syn fibrils at 100 pg/mL or more induced seed-dependent aggregation. In WT mice, α-syn pathologies were detectable at 3 months after intracerebral inoculation of 0.1 μg synthetic mouse α-syn fibrils per animal. The concentration of endogenous α-syn in WT (C57BL6) mouse brain was quantitated and determined to be 37.03 (± 6.71) μg/mL. Previous studies using Tg mice, together with our study, indicate that sensitivity to pathogenic α-syn may vary depending upon the expression level of intracellular α-syn, although we did not use Tg mice in the present work. This is supported by the existence of copy number variations (duplication and triplication) in *SNCA* gene associated with early onset and rapid progression of PD [30]. Overall, these results show that both the amount of pathogenic protein and the concentration of intracellular normal soluble protein are key factors influencing the onset and progression of synucleinopathies. Thus, decreasing the expression level of intracellular soluble α-syn may contribute to suppression of aggregate formation, and inhibit intracerebral propagation.

It has been experimentally investigated whether exposure to pathological α-syn via peripheral routes can induce disease onset and pathology, as is the case for PrP^Sc^. Several reports have shown that peripheral inoculation of synthetic α-syn fibrils or MSA brain homogenates into TgM83 mice can induce α-syn pathologies and lethal CNS disorders. Inoculations into the peritoneal cavity or hindlimb muscle resulted in disease onset with high infectivity compared with inoculation into the tongue [3, 10, 48]. On the other hand, α-syn pathology was not induced in WT mice by inoculation into hindlimb muscle or oral inoculation of even large amounts of synthetic α-syn fibrils [35]. These results may reflect the different expression levels of α-syn in the host animals, as noted above. Thus, the sensitivity to pathogenic proteins may depend strongly on the type of experimental model used. Surprisingly, implantation of stainless steel wires contaminated with MSA brain homogenates into TgM83 hemizygous mice resulted in the development of lethal CNS disorder [65]. In addition, PrP^Sc^ adhering to stainless steel wires has been reported to be more resistant to inactivation than PrP^Sc^ in brain homogenates [19, 44]. These findings imply that there may be a substantial risk of secondary infection from surgical instruments bearing pathogenic α-syn, such as instruments used in deep brain stimulation (DBS) surgery to restore motor function to PD patients. Similarly, stainless steel wires contaminated with amyloid-beta (Aβ) fraction caused amyloidosis in TgAPP23 mice [15]. Furthermore, Jaunmuktane et al. reported that transmission of Aβ seeds though surgical instruments might have occurred in patients who underwent neurosurgery in childhood [28]. Thus, to prevent iatrogenic infection, effective sterilization procedures seem mandatory.

Therefore, we next investigated the efficacy of various commonly used inactivation methods for PrP^Sc^ for abolishing the seeding activity of pathogenic α-syn. Autoclave treatments at 120 °C or 134 °C in the presence of 1% SDS dramatically reduced the seeding activity of pathogenic α-syn, including synthetic fibrils and MSA-syn, in the cultured cell model (Figs. 7c, 9c and Table 1). Further, pathogenic α-syn exposed to a single autoclave treatment at 134 °C did not induce α-syn pathology in WT mouse brain (Fig. 10b and 11). On the other hand, single autoclave treatment at 120 °C, which is commonly used as a general sterilization method, was insufficient to inactivate synthetic α-syn fibrils or MSA-syn (Figs. 7c and 9c). The seeding activity of pathogenic α-syn after boiling treatment for 3 min was almost the same as that of untreated pathogenic α-syn (Figs. 7c, 9c, 10b and 11). Similar heat resistance has been reported for Aβ aggregates and abnormal TDP-43 derived from ALS/frontotemporal lobar degeneration (FTLD) cases [15, 40].

Numerous studies on inactivation of PrP^Sc^ derived from various biological species and strains have been conducted, and have shown that various methods, including strong alkaline agents, protein-denaturing agents, proteolytic enzymes and autoclaving, are effective to abolish infectivity [20]. It has also been reported that the level of inactivation varies depending on the biological species and strain, but combinations of multiple treatments can reliably achieve complete inactivation [16, 59]. Thomzig and colleagues examined the removal of Aβ, tau, and α-syn adhering to medical devices by carrier assay using brain extracts of patients, and treatments with 1 M NaOH at room temperature for 1 h, combined treatments with 0.2% SDS or 0.3% NaOH and autoclaving at 134 °C for 5 min, and treatment with a commercial alkaline cleanser or a hydrogen peroxide solution containing Cu^2+^ were reported to be effective for removal of pathogenic α-syn [60]. On the other hand, formalin-fixed pathogenic Aβ, tau, and α-syn have been reported to retain high seeding activity in vitro and in vivo [17, 29, 50]. Surprisingly, an MSA patient’s brain stored in formalin for over 20 years caused TgM83 hemizygous mice to develop lethal CNS disorder [65]. Thus, the suitability or unsuitability of various inactivation procedures for prion-like proteins is becoming clearer. Overall, PrP^Sc^ and other prion-like proteins seem to show similar responses to inactivation procedures, suggesting these pathogenic proteins have some common structural features. Regarding the handling of synthetic α-syn fibrils in laboratories, Bousset et al. reported that 1% SDS and the commercial cleanser Hellmanex can remove synthetic α-syn fibrils from surfaces of various materials, although they found differences in resistance to 1% SDS among distinct α-syn strains, fibrils and ribbons formed under different physiological conditions in vitro [7]. We also found differences of resistance to inactivation procedures between MSA-syn preparations derived from different cases (Fig. 9c). Thus, it remains important to manage pathogenic α-syn by using appropriate safety cabinets to avoid human exposure. Also, the use of a cup horn-type ultrasonicator is useful to avoid inhalation of aerosols containing fragmented pathogenic α-syn during sample preparation.

## Conclusion

We investigated in detail the prion-like properties of pathogenic α-syn, including synthetic α-syn fibrils, MSA-syn and DLB-syn, using SH-SY5Y cells transiently expressing human WT α-syn and non-Tg mice. We found that synthetic α-syn fibrils and MSA-syn can induce both prion-like amplification of abnormal α-syn in cultured cells and spreading of phosphorylated α-syn pathologies in mouse brain. The seeding activity of MSA-syn is equivalent to or higher than that of synthetic α-syn fibrils, though DLB-syn showed much lower seeding activity. Various combinations of inactivation treatments were effective to abolish the seeding activity of these pathogenic α-syn. Our findings here provide further evidence of the prion-like properties of pathogenic α-syn and re-emphasize the importance of using appropriate inactivation treatments to prevent iatrogenic and secondary infections in the clinical and research fields.

## Additional files

Additional file 1: **Figure S1.** Determination of protein concentration of phosphorylated α-syn in patients’ brains. A, Sarkosyl-insoluble fractions prepared from patients’ brains used in this study were analyzed by immunoblotting with anti-phosphorylated α-syn PSer129 antibody (upper) and anti-tau T46 antibody (lower). B, Standard curve of phosphorylated α-syn, generated by immunoblotting of phosphorylated monomer α-syn. Concentrations of phosphorylated α-syn were determined using this standard curve. Protein concentrations of sarkosyl-insoluble fractions extracted from patients’ brains are shown in **Table S2**. (PDF 139 kb)

Additional file 2: **Table S1.** α-Syn concentrations in sarkosyl-insoluble fractions extracted from patients’ brains, The α-syn concentrations of sarkosyl-insoluble fractions extracted from patients’ brains used for experiments in the cultured cell model (A) and mouse model (B) are shown. (PDF 40 kb)

Additional file 3: **Figure S2.** Seeding activities of serial dilutions of sarkosyl-insoluble fractions from brains of α-synucleinopathy patients, Sarkosyl-insoluble fractions extracted from brains with synucleinopathy patients were diluted and introduced into SH-SY5Y cells transiently expressing human α-syn. Immunoblot analyses of sarkosyl-insoluble fractions (ppt) and sarkosyl-soluble fractions (sup) extracted from cells transfected with serial dilutions of MSA-1(Cb), MSA-2 (FC), MSA-2 (Pu), MSA-3 (FC) and DLB-4 (FC) are shown. Phosphorylated α-syn was detected with anti-phosphorylated α-syn PSer129 antibody. α-Syn was detected with anti-syn 131–140 antibody. Cb: cerebellum, FC: frontal cortex, Pu: putamen. (PDF 283 kb)

Additional file 4: **Figure S3.** Determination of protein concentration of α-syn in C57BL/6 mouse brain, Standard curve of mouse α-syn was generated by immunoblotting of serial dilutions of recombinant mouse α-syn protein. Protein concentrations of endogenous α-syn in mouse brains were determined by interpolation on a standard curve. A68 buffer-soluble fractions were extracted from C57BL/6 mouse brains (*n* = 3). Bands of recombinant proteins and A68 buffer-soluble fractions were detected with anti-mouse α-syn antibody. (PDF 78 kb)

Additional file 5: **Table S2.** Numbers of mice used in experiments. (PDF 52 kb)

Additional file 6: **Figure S4.** Seeding activities of serial dilutions of treated synthetic α-syn fibrils in SH-SY5Y cells, Serial dilutions of synthetic α-syn fibrils exposed to various inactivation treatments were introduced into SH-SY5Y cells. Immunoblot analysis of sarkosyl-insoluble fractions (ppt) and sarkosyl-soluble fractions (sup) extracted from cells transfected with serial dilutions of synthetic α-syn fibrils treated with 1% SDS for 1 h at room temperature, boiling, or autoclaving at 134 °C with or without 1% SDS are shown. Phosphorylated α-syn was detected with anti-phosphorylated α-syn PSer129 antibody. α-Syn was detected with anti-syn 131–140 antibody. (PDF 248 kb)
