# Supplementary figures and images for: Potent prion-like behaviors of pathogenic α-synuclein and evaluation of inactivation methods
Source: Acta Neuropathol Commun. 2018 Apr 18;6:29. doi: 10.1186/s40478-018-0532-2 (PMC5907316; doi:10.1186/s40478-018-0532-2)

Fig S1.

A

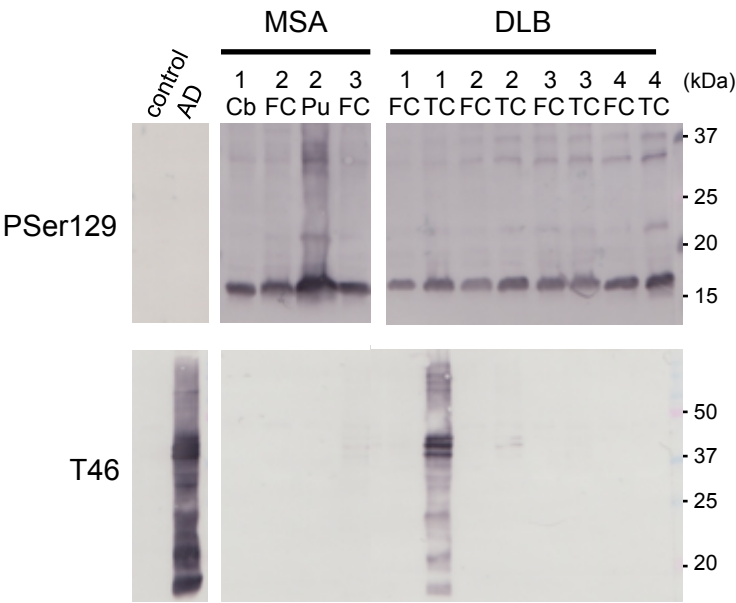

B

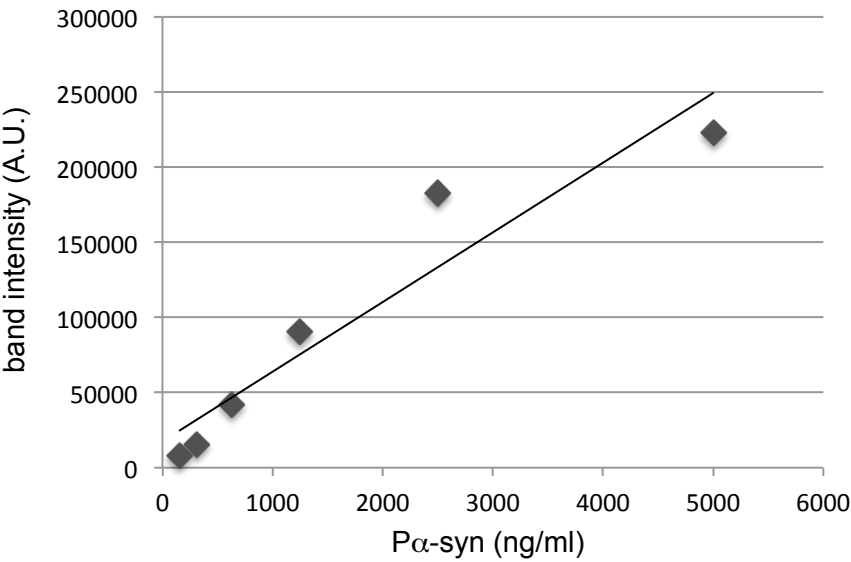

Supplement: Supplementary file 1 — Figure S1. Determination of protein concentration of phosphorylated α-syn in patients’ brains. A, Sarkosyl-insoluble fractions prepared from patients’ brains used in this study were analyzed by immunoblotting with anti-phosphorylated α-syn PSer129 antibody (upper) and anti-tau T46 antibody (lower). B, Standard curve of phosphorylated α-syn, generated by immunoblotting of phosphorylated monomer α-syn. Concentrations of phosphorylated α-syn were determined using this standard curve. Protein concentrations of sarkosyl-insoluble fractions extracted from patients’ brains are shown in Table S2. (PDF 139 kb) [file 40478_2018_532_MOESM1_ESM.pdf]

Fig S2.

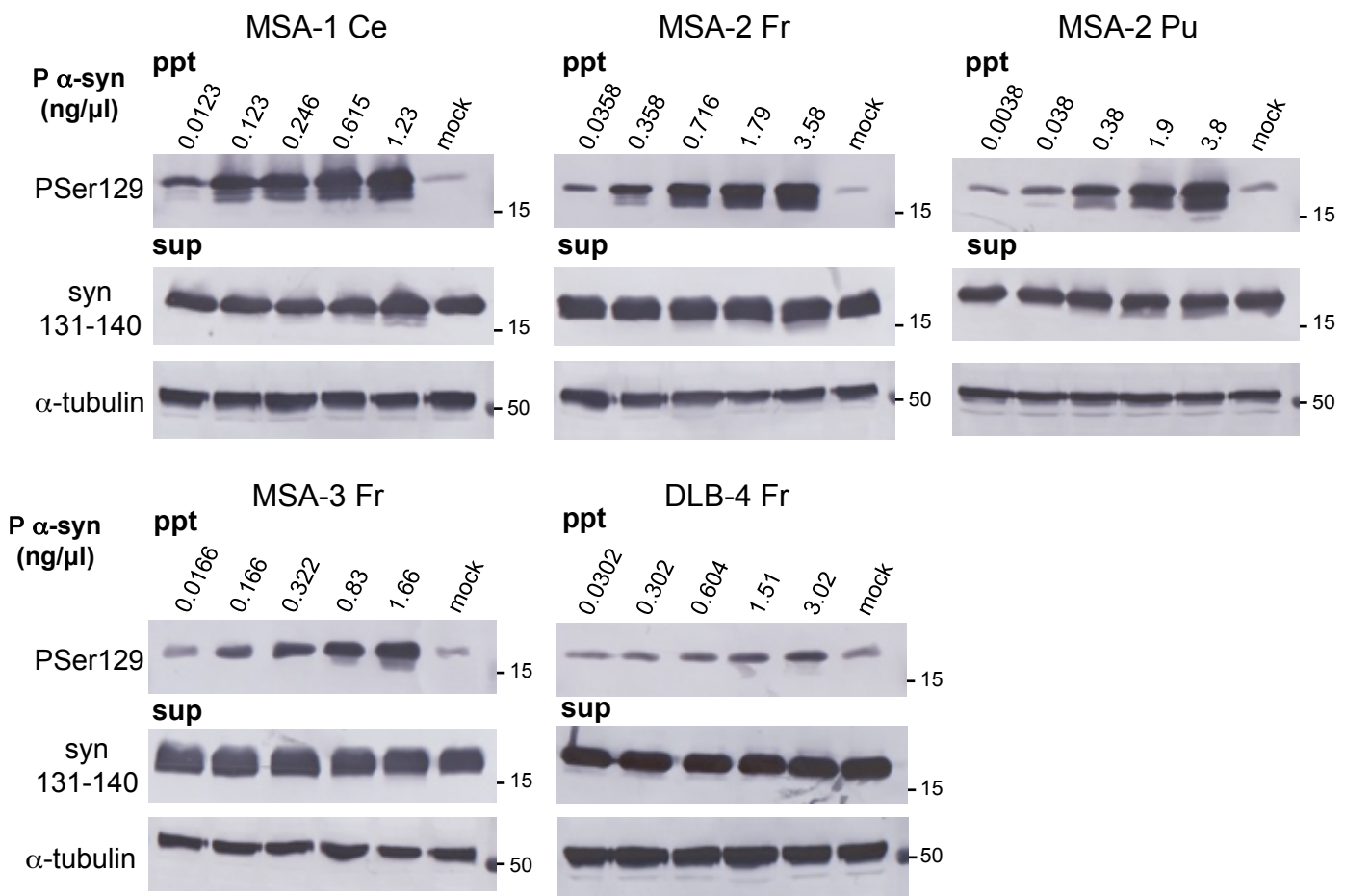

Supplement: Supplementary file 3 — Figure S2. Seeding activities of serial dilutions of sarkosyl-insoluble fractions from brains of α-synucleinopathy patients, Sarkosyl-insoluble fractions extracted from brains with synucleinopathy patients were diluted and introduced into SH-SY5Y cells transiently expressing human α-syn. Immunoblot analyses of sarkosyl-insoluble fractions (ppt) and sarkosyl-soluble fractions (sup) extracted from cells transfected with serial dilutions of MSA-1(Cb), MSA-2 (FC), MSA-2 (Pu), MSA-3 (FC) and DLB-4 (FC) are shown. Phosphorylated α-syn was detected with anti-phosphorylated α-syn PSer129 antibody. α-Syn was detected with anti-syn 131–140 antibody. Cb: cerebellum, FC: frontal cortex, Pu: putamen. (PDF 283 kb) [file 40478_2018_532_MOESM3_ESM.pdf]

Fig S3.

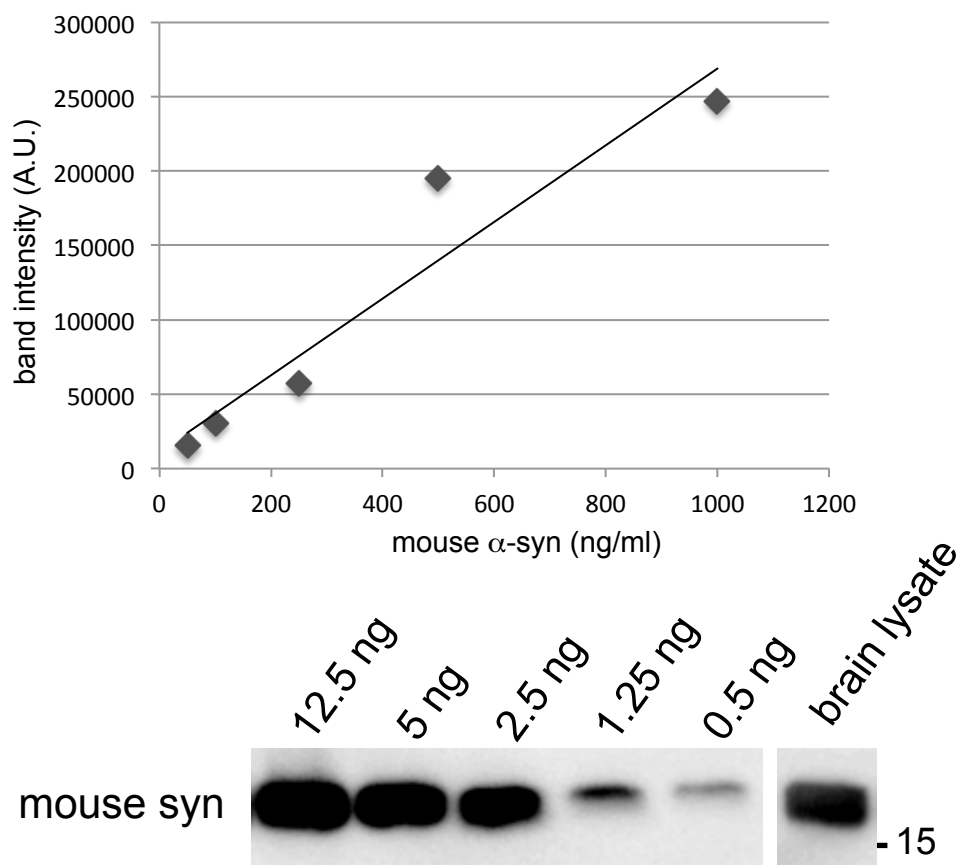

Supplement: Supplementary file 4 — Figure S3. Determination of protein concentration of α-syn in C57BL/6 mouse brain, Standard curve of mouse α-syn was generated by immunoblotting of serial dilutions of recombinant mouse α-syn protein. Protein concentrations of endogenous α-syn in mouse brains were determined by interpolation on a standard curve. A68 buffer-soluble fractions were extracted from C57BL/6 mouse brains (n = 3). Bands of recombinant proteins and A68 buffer-soluble fractions were detected with anti-mouse α-syn antibody. (PDF 78 kb) [file 40478_2018_532_MOESM4_ESM.pdf]

Fig S4.

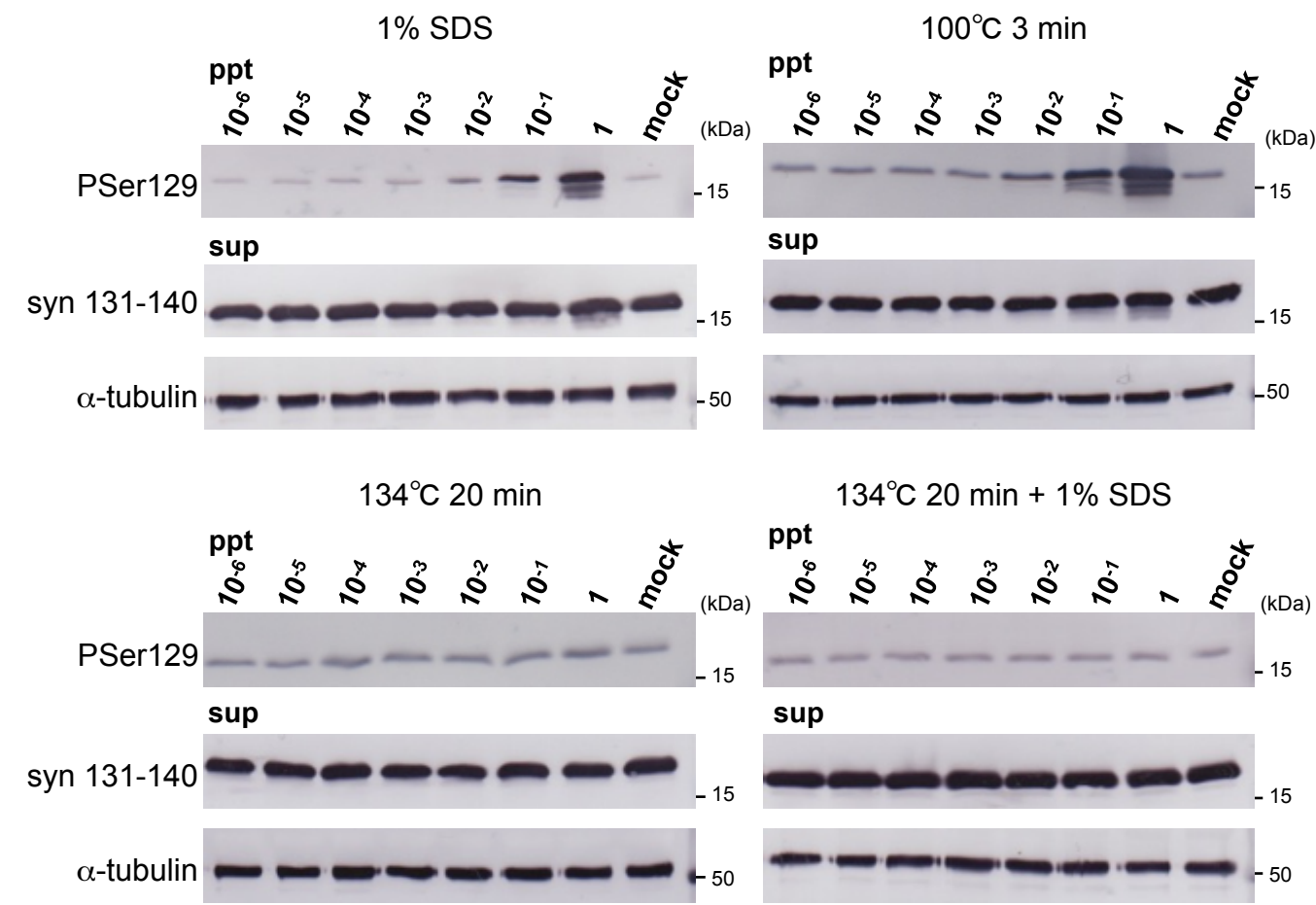

Supplement: Supplementary file 6 — Figure S4. Seeding activities of serial dilutions of treated synthetic α-syn fibrils in SH-SY5Y cells, Serial dilutions of synthetic α-syn fibrils exposed to various inactivation treatments were introduced into SH-SY5Y cells. Immunoblot analysis of sarkosyl-insoluble fractions (ppt) and sarkosyl-soluble fractions (sup) extracted from cells transfected with serial dilutions of synthetic α-syn fibrils treated with 1% SDS for 1 h at room temperature, boiling, or autoclaving at 134 °C with or without 1% SDS are shown. Phosphorylated α-syn was detected with anti-phosphorylated α-syn PSer129 antibody. α-Syn was detected with anti-syn 131–140 antibody. (PDF 248 kb) [file 40478_2018_532_MOESM6_ESM.pdf]
