# Supplementary material for: Potent prion-like behaviors of pathogenic α-synuclein and evaluation of inactivation methods
Source: Acta Neuropathol Commun. 2018 Apr 18;6:29. doi: 10.1186/s40478-018-0532-2 (PMC5907316; doi:10.1186/s40478-018-0532-2)
Supplement: Supplementary file 2 — Table S1. α-Syn concentrations in sarkosyl-insoluble fractions extracted from patients’ brains, The α-syn concentrations of sarkosyl-insoluble fractions extracted from patients’ brains used for experiments in the cultured cell model (A) and mouse model (B) are shown. (PDF 40 kb) [file 40478_2018_532_MOESM2_ESM.pdf]

Table S1.

A

	Pα-syn(ng/ul)
controll	not detected
AD	not detected
MSA-1 Ce	1.23
MSA-2 Fr	3.58
MSA-2 Pu	7.72
MSA-3 Fr	1.66
DLB-1 Fr	1.77
DLB-1 T	1.94
DLB-2 Fr	1.24
DLB-2 T	1.44
DLB-3 Fr	1.11
DLB-3 T	1.33
DLB-4 Fr	1.84
DLB-4 T	1.87

B

	Pα-syn(ng/ul)
control	not detected
MSA-2 Pu	43.6
