# Supplementary material for: Potent prion-like behaviors of pathogenic α-synuclein and evaluation of inactivation methods
Source: Acta Neuropathol Commun. 2018 Apr 18;6:29. doi: 10.1186/s40478-018-0532-2 (PMC5907316; doi:10.1186/s40478-018-0532-2)
Supplement: Supplementary file 5 — Table S2. Numbers of mice used in experiments. (PDF 52 kb) [file 40478_2018_532_MOESM5_ESM.pdf]

# Table S2.

## A

| fibril (μg) | *N* (Total) |
|---|---|
| 40 | 5 |
| 10 | 5 |
| 4 | 4 |
| 2 | 4 |
| 1 | 4 |
| 0.1 | 3 |
| 0.01 (10 ng) | 3 |
| 0.001 (1 ng) | 4 |
| 0.0001 (0.1 ng) | 4 |
| 0.00001 (10 pg) | 4 |

## B

| Treatment | *N* (Total) |
|---|---|
| monomer-saline | 3 |
| saline | 4 |
| 100℃ 3min | 4 |
| 120℃ 20min | 4 |
| 120℃ 20min/0.1% SDS | 3 |
| 134℃ 20min | 3 |
| 134℃ 20min/0.1% SDS | 3 |

## C

| brain samples | *N* (Total) | 3M | 9M |
|---|---|---|---|
| control | 4 | 2 | 2 |
| DLB | 4 | 2 | 2 |
| MSA-2 Pu | 5 | 5 | not tested |
| MSA-2 Pu 100℃3min | 5 | 5 | not tested |
| MSA-2 Pu 134℃20min | 4 | 4 | not tested |
